# Iatrogenic right coronary artery occlusion during minimally invasive cardiac surgery-tricuspid annuloplasty—a case report

**DOI:** 10.1186/s40981-022-00571-y

**Published:** 2022-10-06

**Authors:** Daichi Urabe, Masahiro Ide, Motoyuki Matsuoka, Ryuichiro Miyake

**Affiliations:** 1 Department of Anesthesia and Critical Care, Kobe City Medical Center General Hospital, 2-2-1, Minatojima-minamimachi, Chuo-ku, Kobe, Hyogo 650-0047 Japan; 2 Anesthesia Associates of Kobe, Kobe, Japan; 3 Department of Anesthesia, Akashi Medical Center, Akashi, Japan

**Keywords:** ST elevated myocardial infarction, Minimally invasive cardiac surgery, Intraoperative monitoring, Iatrogenic complication

## Abstract

Although rare, right coronary artery (RCA) injury is a serious complication of tricuspid annuloplasty (TAP) and warrants close attention. We report a case of ST elevation myocardial infarction secondary to iatrogenic RCA occlusion during minimally invasive cardiac surgery (MICS). Electrocardiography (ECG) revealed ST segment elevation in lead II. Transesophageal echocardiography (TEE) revealed inferior wall hypokinesis after cardiopulmonary bypass, and coronary angiography revealed peripheral RCA occlusion. Intraoperatively, we detected an atrioventricular groove deformity during the second surgical procedure. Wall motion and ECG abnormalities showed normalization after TAP was reestablished. Vigilant monitoring using TEE and ECG is important to detect intraoperative myocardial ischemia during MICS-TAP.

**Supplementary Information:**

The online version contains supplementary material available at 10.1186/s40981-022-00571-y.

## Background

Valve repair using minimally invasive surgery involves a smaller surgical incision and is associated with a shorter length of hospitalization and reduced need for blood product transfusion; therefore, this approach is preferred in clinical practice. Although rare, iatrogenic coronary artery injuries during annuloplasty suture placement require careful monitoring owing to limited surgical field visualization available to the surgeon [1]. Anesthesiologists should also be aware of this rare complication and closely monitor the patient’s electrocardiogram (ECG) to promptly identify any hemodynamic changes in arterial pressure and also any abnormalities on transesophageal echocardiography (TEE). We present a case of inferior ST elevation myocardial infarction (STEMI) secondary to iatrogenic right coronary artery (RCA) occlusion during mitral valve repair (MVP) and tricuspid annuloplasty (TAP) using a minimally invasive approach. This manuscript adheres to the applicable EQUATOR guidelines. Written informed consent was obtained from the patient for the publication of this case report.

## Case presentation

**Fig. 1:**
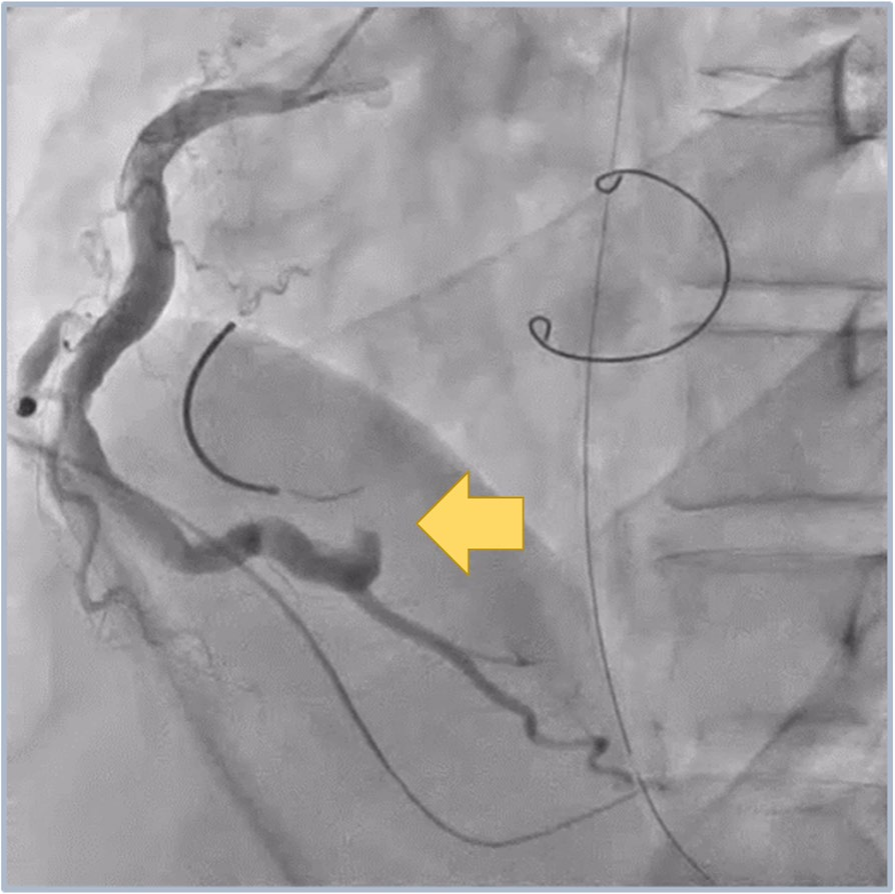
Coronary angiography after tricuspid annuloplasty by minimally invasive cardiac surgery. It reveals the absence of the posterior descending branch of the RCA (arrow), with intact other branches

**Fig. 2:**
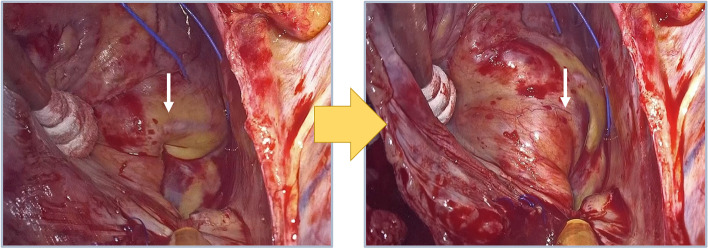
Atrioventricular sulcus of the right ventricle before (left) and after (right) reoperation. Note a dimple in the atrioventricular sulcus, which disappeared after reoperation (arrows)

A 44-year-old man underwent minimally invasive cardiac surgery (MICS), MVP, and TAP. He had a medical history of hypertension and angina pectoris and received percutaneous coronary intervention (PCI) for significant left descending artery stenosis. Preoperative transthoracic echocardiography (TTE) revealed severe mitral valve regurgitation secondary to mitral annular dilatation with posterior leaflet prolapse, mild tricuspid regurgitation secondary to tricuspid annular dilatation, and slightly reduced left ventricular systolic function with an ejection fraction of 59% without regional wall motion abnormalities. Preoperative coronary angiography revealed no significant stenosis. General anesthesia was induced using thiamylal, fentanyl, and rocuronium and was maintained using air-oxygen-sevoflurane and remifentanil. Sevoflurane administration was switched to continuous propofol infusion during cardiopulmonary bypass (CPB). We performed tracheal intubation using a 37-Fr left-sided double-lumen tube (Broncho-Cath®, Dublin, Ireland). The ultrasound-guided paravertebral block was performed in the right fourth paravertebral space using 20 ml of 0.25% levobupivacaine with catheter placement (Standard Perifix®, B-Braun, Hessen, Germany). Comprehensive intraoperative TEE evaluation (EPIC®, Phillips Medical Systems, MA, USA) revealed mitral valve prolapse of the P2 and P3 regions of the mitral valve leaflets along with annular dilatation. Following the right 4th mini-thoracotomy and establishment of CPB, we achieved cardiac arrest using antegrade myocardial protection (Myotector®, Kyowa Criticare Co., Ltd., Tokyo, Japan), which was infused every 30 min to maintain a cardiac standstill. We performed successful chordal reconstruction for mitral valve repair and annuloplasty ring and TAP. Unclamping the aorta led to immediate restoration of sinus rhythm, and the patient was weaned from CPB without administration of inotropic agents. ECG showed ST segment elevation in lead II, and TEE revealed severe hypokinesis of the inferior left ventricular wall (Supplementary Video). Although the patient’s blood pressure was maintained at a high level for better perfusion for suspected temporary air embolization into the RCA, ST segment elevation worsened but without hemodynamic deterioration. Postoperatively, we observed ECG changes corresponding to the entire inferior wall and not only in areas corresponding to lead II, with reciprocal changes in lead I and the anterior leads (V1–V4). The patient was immediately transferred to the catheterization laboratory and underwent coronary angiography, which revealed disruption in the flow of contrast media distal to the posterior descending branch of the RCA (#4PD) (Fig. 1). Emergency PCI was attempted; however, the catheter guidewire failed to pass through the distal RCA. TAP-induced mechanical occlusion of the RCA was suspected, and the patient was retransferred to the operation theater for repeat MICS-TAP to release the obstruction. Following re-establishment of CPB and cardiac arrest using the same cardioplegic solution administered during the previous operation, careful inspection revealed a dimple in the interatrial sulcus (Fig. 2). The dimple disappeared following removal of the prosthetic annuloplasty ring sutures. Immediate coronary angiography confirmed clearing of the occlusion of the descending branch of the RCA, and we performed successful TAP. The patient was hemodynamically stable and was easily weaned off CPB without inotropic support. The ST segment returned to normal upon completion of surgery. The patient was extubated shortly after admission to the intensive care unit. His serum maximum creatine kinase level was 6795 IU/l, and the creatine kinase MB protein fraction measured 573 IU/l, both of which normalized on postoperative day 3. Postoperative follow-up TTE revealed no regional wall motion abnormalities, and the patient was discharged on postoperative day 9.

## Discussion

Intraoperative myocardial ischemia secondary to left circumflex artery injury is a well-known complication of mitral valve surgery [1]. RCA injuries associated with tricuspid valve surgery are rare [2–4]. However, the RCA is separated from the tricuspid annulus by only a few millimeters; therefore, myocardial ischemia secondary to mechanical RCA obstruction or injury may occur during TAP [5]. Suture-induced deformity of the tricuspid annulus should be suspected as a cause of RCA injury during MICS-TAP [6].

Intraoperative ECG and TEE play a key role in the early detection of myocardial ischemia during cardiac surgery, particularly MICS, in which optimal visualization of the surgical field is limited. Previous studies have reported ST changes, arrhythmias, and right-sided heart failure with tricuspid regurgitation as signs of myocardial ischemia in hemodynamically stable patients [3, 6], which suggests that anesthesiologists should be vigilant regarding ECG and TEE alterations, particularly during MICS. In our patient, the suture-induced dimple at the interatrial sulcus resulted in RCA occlusion, which improved rapidly after removal of the annuloplasty suture and avoidance of conversion from lateral mini-thoracotomy to median sternotomy for coronary artery bypass grafting. PCI may serve as a promising therapeutic approach for stenotic lesions; however, this strategy may be unsuccessful in cases of complete mechanical obstruction, as described earlier.

Myocardial ischemia attributable to coronary air embolization or spasms is reversible and usually resolves within a short period if adequate coronary perfusion is maintained. ECG evidence of ST-segment elevation in lead II immediately after weaning off CPB in our patient initially led to a high index of clinical suspicion for reversible myocardial ischemia. However, TEE did not reveal normalization of wall motion abnormalities and ECG changes lasted despite the patient’s hemodynamically stable status. Unfortunately, owing to the patient’s hemodynamic stability and our inability to detect the dimple-induced RCA occlusion, we were unable to accurately diagnose this condition during the first operation, which led to a delay in performing coronary angiography and repeat thoracotomy, which resulted in prolonged myocardial ischemia. Early detection and accurate diagnosis of intraoperative myocardial ischemia using ECG and TEE and being mindful of the possible causes are important to select the most appropriate treatment and avoid unnecessary interventions [7].

In conclusion, intraoperative iatrogenic RCA occlusion may occur during MICS-TAP; continuous monitoring using ECG and TEE is useful for accurate diagnosis of this life-threatening complication to facilitate the timely initiation of appropriate treatment for intraoperative myocardial ischemia.

## Supplementary Information

**Additional file 1: Video S1.** The TEE image, at transgastric mid-papillary short axis view, revealed severe hypokinesis of the inferior wall of the left ventricle.

## Data Availability

Not applicable.

## Abbreviations

TAP: Tricuspid annuloplasty
RCA: Right coronary artery
MICS: Minimally invasive cardiac surgery
ECG: Electrocardiogram
TEE: Transesophageal echocardiography
STEMI: ST elevation myocardial infarction
MVP: Mitral valve repair
PCI: Percutaneous coronary intervention
CPB: Cardiopulmonary bypass
TTE: Transthoracic echocardiography
